# The microbiota as a modulator of mucosal inflammation and HIV/HPV pathogenesis: From association to causation

**DOI:** 10.3389/fimmu.2023.1072655

**Published:** 2023-01-23

**Authors:** Elena Moreno, Raquel Ron, Sergio Serrano-Villar

**Affiliations:** ^1^ Department of Infectious Diseases, Hospital Universitario Ramón y Cajal, Facultad de Medicina, Universidad de Alcalá, IRYCIS, Madrid, Spain; ^2^ CIBERINFEC, Instituto de Salud Carlos III, Madrid, Spain

**Keywords:** microbiota, HIV, HPV, inflammation, biomarkers, omics, personalized medicine

## Abstract

Although the microbiota has largely been associated with the pathogenesis of viral infections, most studies using omics techniques are correlational and hypothesis-generating. The mechanisms affecting the immune responses to viral infections are still being fully understood. Here we focus on the two most important sexually transmitted persistent viruses, HPV and HIV. Sophisticated omics techniques are boosting our ability to understand microbiota-pathogen-host interactions from a functional perspective by surveying the host and bacterial protein and metabolite production using systems biology approaches. However, while these strategies have allowed describing interaction networks to identify potential novel microbiota-associated biomarkers or therapeutic targets to prevent or treat infectious diseases, the analyses are typically based on highly dimensional datasets —thousands of features in small cohorts of patients—. As a result, we are far from getting to their clinical use. Here we provide a broad overview of how the microbiota influences the immune responses to HIV and HPV disease. Furthermore, we highlight experimental approaches to understand better the microbiota-host-virus interactions that might increase our potential to identify biomarkers and therapeutic agents with clinical applications.

## 1 Introduction

Evolutionary and ecological mechanisms have favored the cooperation of microorganisms that ensure critical functions for host fitness, such as the response against viral infections. The largest fraction of the microbiota resides in close interaction with the mucosa-associated lymphoid tissue (MALT) (1). Therefore, the expectations that the microbiota could exert a clinically relevant impact on viral infections, such as HPV and HIV, are high. The pathogenesis of HPV and HIV infection is intimately associated with the MALT, from the early establishment of infection to their persistence or progression (2–4). For example, HIV infection causes chronic defects in mucosal immunity (5, 6) and translocation of microbial products from the gut to the blood. These changes promote T cell activation, monocyte activation, and proinflammatory cytokine release (7–10). In HPV, the gut microbiota appears to influence viral persistence, immune responses, the host-mucosal environment, and HPV-related cancer progression (11, 12).

Although omics technologies have allowed us to map the functional alterations produced by viral infections, many studies show correlations, and we lack a granular understanding of the underlying mechanisms for the functional alterations. Omics techniques have allowed linking specific microbiome profiles to certain disease phenotypes (13, 14). The influence of bacterial proteins and metabolites on disease is gaining interest and being more deeply studied (15–21). However, most studies in the field are still correlational and hypothesis-generating. Furthermore, although proteomics and metabolomics are helpful tools to infer pathways and generate hypotheses and they have become increasingly efficient, their results still have limitations and biases and warrant experimental validation. Thus, following the enthusiasm of omics-based studies, the classical approach of designing hypothesis-driven studies focused on digging deeper into particular questions after interrogating highly dimensional datasets, is gaining attention. Here, we review specifically the current concepts on the reciprocal interactions between the microbiota and two persistent viral infections, HIV and HPV. We discuss the opportunities for omics techniques and their limitations in the field, highlight examples of studies aimed at understanding the consequences of the microbiota in HIV and HPV infections, and summarize the experimental approaches that have improved our mechanistic insight.

## 2 Influence of the microbiota on HIV and HPV infections

Correlations between changes in gut mucosa leading to “dysbiosis” (i.e., alterations in the intestinal microbiota) and viral infections are commonly studied. The commensal microbiota appears to be a significant determinant of the acquisition and replication of some pathogenic viruses. This may include mechanisms not well understood yet, including pathogen growth regulation, competitive metabolic interactions, localization in intestinal niches, and host-immune response induction (22–26). However, as we will review below, there is a clear connection between the microbiota status and the clinical course of HIV and HPV.

### 2.1 Influence of HIV infection on the microbiota

Acute HIV infection exerts dramatic and perhaps irreversible MALT damage (27, 28). The fact that HIV replication has been associated with a loss of anti-inflammatory bacteria (20, 29) has spurred research into the hypothesis that HIV infection affects the microbiota and that this altered microbiota may contribute to persistent inflammation, increasing the risk of comorbidities. Mechanistically, HIV infection could affect the microbiota by inducing depletion of Th17 cells in MALT, enteropathy, mucosal inflammation, aberrant cytokine production, and intestinal epithelial cell damage (25, 30–34). In the context of HIV infection, microbial-induced immune activation occurs and correlates with markers of intestinal damage, suggesting that the microbiota is a relevant driver of systemic inflammation (35–41). Even the changes appreciated in the oral microbiota of people living with HIV (PLWH), who exhibit an increased prevalence of dental caries and periodontal inflammation, seem to be connected to shifts in systemic immune responses (reviewed in (42)). Specific Lactobacillus species-rich vaginal microbiota have been associated to protection from HIV infection (last reviewed in (43)).

It is now widely accepted that impairment of intestinal integrity and dysbiosis lead to translocation of bacterial derivatives from the gut to the bloodstream, resulting in chronic inflammation. This may occur by immunosuppressive or immunostimulatory mechanisms and *via* various non-mutually exclusive processes, including augmented antigenicity, adjuvanticity, or bystander T-cell activation (44, 45). This fact has been studied before for HIV-associated inflammation, which has been associated with an increase of active microorganisms leading to different pathways related to immune modification (46), These pathways include (i) decreased amino acid catabolism, leading to nutritional deficits (47). (ii) induction of indolamine-2,3-dioxygenase-1 (IDO1) leading to an increased transformation of tryptophan into the immunosuppressive kynurenine derivatives, bacterial translocation, and systemic inflammation, which has been linked with excess mortality risk ,^45^). (iii) increased butyrate synthesis, which, among other functions, tempers intestinal inflammation (48). and (iv) accumulation of inflammatory molecules, such as arachidonic acid and leukotriene-B4 (49).

Inflammatory biomarkers levels remain increased in PLWH even when ART is started early (50). Chronic inflammation has consistently been associated with an excess risk of comorbidities during treated HIV infection and is suggested as a contributing risk factor (51, 52). Thus, the HIV field has pursued whether the microbiota affects inflammation during treated infection. For example, microbiota metabolic profiles affect HIV inflammation by promoting changes in glutathione metabolism and zeatin biosynthesis, butyrate production, or tryptophan catabolism (46, 49, 50, 53). Furthermore, a well-defined deleterious consequence of HIV infection is bacterial translocation triggering immune activation (54–56). A few sequence-based and ultramicroscopic studies have uncovered a blood bacterial DNA profile in HIV. Following acute SIV infection in macaques, analysis of bacterial DNA isolated from the colon, liver, and mesenteric lymph nodes demonstrated a preference for the phylum Proteobacteria to translocate to these compartments and an increased metabolic activity of Proteobacteria within the colonic lumen (57). In a study in PLWH diagnosed with advanced disease and starting ART, we also found that Proteobacteria was the predominant phylum in the blood, indicating commonalities in the mechanisms by which bacterial translocate from the gut into the bloodstream between SIV and HIV infections. The same study showed that ART initiation in late-presenters attenuated the bacterial signature of untreated HIV infection, characterized by the presence of DNA from commensal bacteria with pathogenic potential (58). Relationships between the translocated microbiome, systemic inflammation, and clinical outcomes were described in a different study showing increased CD4 T cell counts following one year of ART that were associated with high *Serratia* abundance, innate proinflammatory cytokines and metabolites driving Th17 gene expression signatures, and restoration of mucosal immunity (59).

Current evidence supports investigating therapeutic strategies for immune modulation in HIV. However, so far, no intervention targeting the microbiota of PLWH either by using prebiotics (16, 16, 60), probiotics (61–63), synbiotics (64), rifaximin (65, 66), or even fecal microbiota transplants (67, 68) have convincingly proved to effectively temper inflammation or enhance boost immune recovery following ART initiation. In general, there was lack of standardization in the outcomes assessed (ranging from studies designed to assess T cell changes (60, 64) to exploratory studies evaluating multiple markers of T cell activation (69) or soluble markers of inflammation or bacterial translocation), the duration of the intervention (from weeks (16, 61, 62, 69) up to one year), the disease status (from ART naive patients followed without ART (60) or patients presenting at advanced stages of the disease (64) starting ART to patients under ART-mediated HIV RNA suppression (61–63), and even the dosage and components of the prebiotic or probiotic mixtures (16, 62–64, 69). Therefore, this still represents a field of active research, and has been extensively reviewed elsewhere (70).

### 2.2 Influence of HPV infection on the microbiota

We know less about the impact of HPV infection on the microbiota epithelial surface integrity, mucosal state, and immune regulation, all factors related to HPV persistence and progression to cancer (46, 71–74). For example, metabolites associated with the vaginal microbiome, including biogenic amines, glutathione, and lipids, have been implicated in HPV persistence (75). It has been described that the microbiota composition can affect all these factors in the context of HPV infection (76–80). A meta-analysis found that *Lactobacillus iners* and non-*Lactobacilli* species dominance in the vaginal microbiota is associated with a higher risk of persistent HPV infection and dysplasia (22) compared to the dominance of *L. iners* and L. c*rispatus* (81, 82).

A *Lactobacillus-depleted* microbiome has been associated with a proinflammatory environment that may increase malignant cell proliferation and HPV E6 and E7 oncogene expression (22, 83, 84) and promote coinfections by other pathogens such as *Chlamydia trachomatis* (80). Specifically, it has been shown that HPV down-regulates some innate molecules, such as SLPI, S100A7, elafin, HβD1, and TNFα/LPS that are used by some *Lactobacillus* species as an amino acid source sustaining their growth, in keeping with their decreased abundance in microbiome analyses of HPV infected individuals (80). Even virome alterations are associated with features of the vaginal microbiota and genital inflammation changes related to HPV infection (85).

Some authors have connected the expression of proinflammatory and chemotactic cytokines related to HPV-induced carcinogenesis with an increased presence of *Sneathia* or *Gardnerella* in the vaginal microenvironment (86–90). Furthermore, even though a certain level of inflammation has been described as potentially beneficial to decrease HPV dissemination, several studies have shown specific inflammation markers as related to the progression to a carcinogenic status that could be used as clinical markers to prevent high-grade squamous intraepithelial lesions (46, 86, 90–95) and specific metabolic profiles (96–98). However, most studies in the field are cross-sectional, so it is hard to assess whether the microbiota influences HPV infection or vice versa.

### 2.3 Microbiota mechanisms with consequences on viral infection

Viruses infecting epithelial cells can profoundly affect the mucosal immune system—the central habitat of the mucosal microbiota—altering the immunological signals required to orchestrate commensal colonization and possibly affecting systemic immune responses and other processes. While from an applied perspective, the gut microbiota functionalities are more relevant for health, most studies have focused on the compositional level, and only fewer studies have focused on the functional consequences. Some microbiota-associated mechanisms possibly influencing the clinical course have been characterized for HIV and HPV (**Table 1**).

**Table 1:** Summary of the major mechanisms by which the microbiota influences HIV and HPV infection.

Pathway/Function	Virus	Bacteria implicated	Biological Mechanism	Clinical Consequences	References
Regulation of innate immune molecules	HIV	↓ *Lactobacilli* *↓ Lachnospira* spp. *↓ Roseburia intestinalis* *↓* Ruminococcaceae	Peptidoglycan signaling Decreased butyrate production Increased local inflammation	Increased HIV transmission	(16, 99–101)
	HPV	↓ *Lactobacilli* ↓ *Bifidobacterium* *↑ Anaerobes and diversity*	Down-regulation of SLPI, S100A7, elafin, HβD1, TNFα/LPS. Cytokines and chemokines	Enhance antitumor immunity and anti-PD-L1 efficacy. Higher risk of sexually transmitted infections	(102–105)
Tryptophan catabolism	HIV	*↑ Gammaproteobacteria* *↑ Pseudomonas* spp. *↑ Bacillus* spp. *↑ Burkholderia* spp. *↑ Prevotella* *↑ Acidaminococcus*	Immunotolerance Barrier failure Angiogenesis IDO1 inhibition ↑ immunosuppressive kynurenine derivatives ↓ Th17 cells Bacterial translocation	Higher risk of non-AIDS comorbidities	(15, 53, 106, 107)
	HPV	*N.f.*	Increased kynurenine derivatives increase oxidative stress	HPV malignant transformation to cancer	(108)
IL-10 signaling pathway	HIV	*↑ Bacteroides fragilis*	Immunotolerance: Polysaccharide A production TLR-2 activation IL-10 expression Systemic immune activation. Inflammation	Periodontitis Higher risk of non-AIDS comorbidities	(53, 109–112)
	HPV	↓ *Lactobacilli*	IL-10 increase breaks the balance with IL-2 leading to Th2 dominance	Immunosuppression state leading to progress of lesions	(113)
Choline metabolism	HIV	↑ Actinobacteria ↓ Bacteroidetes ↑ Firmicutes ↑ Gammaproteobacteria ↑ Clostridium XIVa ↑ *Faecalibacterium* spp.	Endothelial dysfunction Inflammation. TMAO production. Monocyte activation	Increased atherosclerosis and cardiovascular risk	(114–116)
	HPV	*-*	Aberrant DNA methylation associated with HPV infection.	Cervical tumorigenesis	(117)
Activation of adaptative immunity	HIV	↑ Bifidobacteria	CTL responses Epithelial cell turnover Immunomodulatory strain-dependent effects ↑ Dendritic cell activation ↑ CD8+ T cell priming and accumulation in the tumor microenvironment ↑ Cross-reactivity with tumor antigens	Improved immune recovery under ART	(118, 119)
	HPV	↓ *Lactobacillus dominance* *↑ Anaerobes and diversity*	Recruitment of immune cells	Bacterial vaginosis (BV)	(120)
Chemotaxis	HIV	↓ *Akkermansia muciniphila*	Host immune regulation ↓ Mucin degradation. Higher systemic inflammation (sCD14, IP10) and intestinal inflammation (fecal calprotectin)	Higher risk of non-AIDS comorbidities	(121, 122)
	HPV	↓ *Lactobacillus dominance* *↑ Anaerobes and diversity*	Reduction in the viscosity of the cervicovaginal fluid (CVF), due to the production of mucin-degrading enzymes	Breaking the first line of defense against exogenous pathogen colonization.	(120)
Cell proliferation	HIV	*↑ Fusobacterium* spp.	Cell proliferation and oncogenesis: TLR-4 signaling. PPAK1 cascade. Nuclear factor KB induction	Impaired immune recovery after ART	(29, 123)
	HPV	*↑ Lactobacillus inners* *↑ Gardnerella vaginalis* *↑ Atopobium vaginae* *↑ Sneathia* *↑ Fusobacterium* *↑ Chlamydia trachomatis*	Persistent coinfection with other bacteria is linked to epigenetic changes, oncogenes expression, non-coding RNA regulations, p53 deregulation, etc. But no direct experimental evidence for bacteria other than *C. thrachomatis* and found LPS from bacteria in exosomes	Association with cervical intraepithelial neoplasia (CIN) to carcinoma *in situ* (CIS)	(124)
Inflammation. Antitumoral immunity	HIV	↑ *Lactobacillales*	Enhanced antitumor response: Upregulated IFN-γ, GZMB, and PRF1 expression in CD8+ T-cells	Improved immune recovery after ART	(20, 123, 125)
	HPV	–	The interplay of viral oncoproteins and inflammatory cytokines leads to continuous immune evasion, which promotes the progression of the lesion. Also, increased oxidative stress has been attributed to inflammation	Progression of the initial lesion to malignancy	(126–129)

↑ increased abundance and ↓ decreased abundance.

## 3 Potential and limitations of current approaches for understanding microbiota effects on HIV and HPV infections

Studying the interactions between host factors and pathogens is complex, especially when a third term—a virus— is added to the multifaceted dichotomy of host and microbiota. However, multi-omic techniques have allowed applying systems biology approaches and ecological concepts to analyze host-microbiota interactions during viral infections (130, 131). These approaches have boosted our ability to understand viral infection to the level in which we are starting to appreciate the importance of the commensal bacterial communities on the pathogenesis of diseases that not so long ago were assumed to depend only on the interactions between viruses and human cells. Nevertheless, current omic techniques have several limitations, such as the scarcity of standardized methods to integrate the different omic levels (132). More importantly, the research potential and fascination with the increasingly efficient omics approaches have often relegated hypothesis-driven research to a second position. We believe that, while the microbiome research primarily relying on 16S rRNA gene studies has been crucial to generate hypotheses, the field needs to move towards more mechanistic, hypothesis-driven studies and applied research.

### 3.1 Multiomic techniques

Technologies such as Next Generation Sequencing (NGS), RNA sequencing (RNA-seq), and mass spectrometry (MS/MS) and all their different variations have already been used to describe the global landscape of viral-host interactions. Typically, gain and loss-of-function studies are performed to study the differential expression of DNA or RNA, either of human or bacterial origin, after viral infections. However, since this cannot capture the whole picture of the complex interactions between the virus and the host, mass spectrometry started being used to study the complete proteome, secretome, and metabolome, and even for bacterial identification in clinical microbiology (133). In addition, some studies have used these technologies, even performing integration of some of them (134), to study the role of the microbiome in the inflammation state produced by HIV infection (20) and reviewed in (24, 46, 135) and in pathogenesis and progression to cancer after HPV infection (74, 93, 136).

Improvements in meta-omic techniques have mainly been used to study the totality of the aimed compounds (genes, proteins, and metabolites) in a set of commensal organisms (metagenomics, metaproteomics, and metametabolomics). Currently, sophisticated versions of these methodologies are becoming more commonly used. For example, shallow metagenomics sequencing is being used to obtain strain-level resolution (137). This, together with the development of advanced computational methods (138), is increasing our resolution allowing the identification of novel strains with probiotic potential, an unmet need by previous studies with prebiotics or probiotics in PLWH (39, 64). Other thriving methods include single-cell technologies, which allow isolating, culturing, and characterizing the genomes and transcriptomes of individual microbes in complex communities (139), or tridimensional mapping of the host microbiota interactions within the mucosa, which is advancing our understanding of the microbiota-immune response interactions to the next level (140).

### 3.2 From hypothesis-generating microbiota studies to hypothesis-driven and applied research

Inside and outside the HIV and HPV fields, the lack of methodological standardization is one of the main limitations in the study of the microbiome and challenges reproducibility (141). For example, a comparison of the clinical impacts of the use of probiotic showed very different results (142) (see **Table 2**). Although, as discussed before, technologies are improving, and now is possible to perform whole genome shotgun sequencing to enhance the detection of diversity, prediction of genes, and accuracy of bacterial species detection (166). However, it is also important to complement the studies by using omics other than genomics to obtain information at the functional level, although there are also challenges regarding the standardization of these methodologies (136, 167, 168). One of these challenges is the integration of datasets (169), which has led to the proposal of the use of machine learning and artificial intelligence for this task, which also have intrinsic limitations (170).

**Table 2:** Summary of the experimental models used to study the effects of microbiota on HIV and HPV infection.

Virus	Experimental model	Mechanism identified	References
HIV	Immune cells stimulation with fecal bacterial communities isolated from HIV patients	Enteric microbiota of untreated HIV-infected subjects induces monocytes and T-cell activation.	(41, 143)
	Immune cells stimulation with LPS from specific bacteria, such as mycobacteria or *Holdemanella*, related to HIV infection	Chemokines and IL-1β released by macrophages. T-cell activation. Macrophages tolerance. Higher frequency of CCR5+CD4+T cells.	(41, 144–150)
	Effect of fecal microbial transplantation on immunity-related to HIV	Increased Th17 and Th22 cells and reduced CD4+Tcell activation.	(67, 68, 151, 152)
	Study of immune activation after fecal transplant in gnotobiotic mice of feces from HIV-negative vs HIV-positive individuals	Non-significant differences	(143)
	Treatment of infection with extracellular vesicles (EVs) or outer membrane vesicles (OMVs) derived from bacteria such as Lactobacilli or Neisseria meningitidis	Demonstrated direct interaction of EVs with viral proteins	(153–157)
	Characterization *in vitro* of the anti-HIV properties of differentially detected candidates by metabolomics	Dipeptides bind to HIV, acting as antivirals and supporting Prevotella growth.	(158)
HPV	OMVs containing HPV antigens create an antitumor vaccine	OMVs stimulated the expression of dendritic cell maturation markers and interferon-gamma-expressing splenocytes.	(157, 159, 160)
	Quantification of bacterial release from vaginal swabs	Differential results depending on the used swab	(161–163)
	Coinfections of bacteria, protozoan, and viruses and quantification of inflammatory cytokines	Galectin-mediated immunity dysregulation	(164)
	Three-dimensional cervical epithelial cell model to study bacterial vaginosis	Identification of some metabolites acting as inflammatory mediators	(165)

Omic technologies result in compositional profiles and large taxonomic lists for which we lack culture methods in most cases. ‘Culturomics’—a high-throughput culture method— and MALDI-TOF mass spectrometry allow the growth of fastidious bacteria together with the identification of several bacterial species and longer incubation periods. However, these techniques have only allowed us to partially overcome the previously mentioned limitations (171). Furthermore, validation of results obtained from the omic techniques is challenging since, in most of the cases, if validation is performed, only a few of the most statistically significant hits are selected for validation. Even when results are validated, any assumption made or reductionist approach used in the experimental design need to be revisited in order to ensure that the results are physiologically relevant and translation to their clinical use can be performed.

In the case of microbiota studies, omic techniques may often result in compositional profiles and large taxonomic lists for which we lack culture methods in most cases. ‘Culturomics’—a high-throughput culture method—allows the growth of fastidious bacteria and more extended incubation periods, and MALDI-TOF mass spectrometry allows the identification of several bacterial species. However, these techniques have only allowed us to partially overcome the previously mentioned limitations (171).

Thus, the previously described shortcomings pose an enormous challenge to unleashing the clinical potential of microbiota role in medicine. If we want to assess the causal-effect relationship better and move towards applied microbiome research, it will be necessary to start with a clinical question and use the most consistent methodology to perform hypothesis-driven research that identifies convincing interactions and confounders. For this, we will need to define first the best hypothesis inspired by a clinical question. Then, from the hypothesis, we should carefully design the experimental approaches (e.g. different omics) and analysis (e.g. network models) and further perform experimental validation, including controls and complementary data sets (e.g., qPCR to confirm sequencing, immunoblot to confirm proteomic, fluorescence resonance energy transfer to confirm AP-MS data, infection kinetics, results validation in external cohorts, etc).

For example, we recently sought to solve a clinical need using applied microbiome research. We asked whether the microbiome could be harnessed to improve the prevention of anal precancer—a leading neoplasia in PWLH— for which we need better screening tools. After investigating a discovery and a validation cohort of at-risk patients, we discovered twelve proteins, previously reported to be associated with cancer progression, that were overexpressed in the anal bacteria from subjects with precancerous lesions. Since these proteins contribute to succinyl-CoA and cobalamin production, we measured the intracellular bacterial concentrations of these metabolites. We discovered that cobalamin and succinyl-CoA were increased in the anal microbiome of patients with anal precancer and overperformed the reference test—anal cytology—. Furthermore, we validated the findings in an external validation cohort, and we demonstrated greater *in vitro* production of succinyl-CoA and cobalamin in bacteria associated with HSIL or cancer vs. those presumably protective (172). Therefore, starting from a clinical question and integrating data from different omic levels we were able to define a new microbiome-based tool that could help in the prevention of a common cancer in PLWH by discovering two powerful biomarkers of anal precancer that could improve anal cancer prevention.

## 4 Experimental models commonly used to study the effects of microbiota on HIV and HPV infection

To overcome the limitations mentioned before and demonstrate the mechanisms driving the effects of the microbiota on HIV and HPV infection, hypothesis-driven experimental designs based on the information generated from the omics technologies should be encouraged. Some leading studies using this approach have been performed in HIV and HPV fields and are summarized in **Table 2**.

Although some improvements are being established in the experimental designs to demonstrate mechanisms led by microbiota components, there are still several limitations. These include a lack of standardization of the methods for obtaining the samples; understanding of differences on the effect of microbiota compartments (such as feces, tissues or EVs) and finding their correct origin (173); or extrapolation of findings in other model organisms, such as rodents (174), to human diseases, that are unrealistic. Furthermore, the *in vivo* models have been helpful in the past in proving the functional consequences of the microbiota. However, the differences between the animal models and the human anatomy, immune system, and genetic background are significant, and the type and mechanisms of interactions of the host with the pathogens are hard to reproduce, even in humanized mice models. Even more, if we only look at studies based on the human model, we still find difficulties in setting up proper validations and standardizations. For example, a significant challenge for human studies is controlling for confounding factors beyond age, sex, and sexual preferences (175), such as host genetic, diet, life style or presence of other pathologies or infections.

## 5 Future perspectives

Although in the last decade, we have witnessed remarkable advances in the field of the microbiota in HIV and HPV infections, we still need to improve our understanding of the specific mechanisms by which the microbiota influences HIV and HPV pathogenesis and how effectively modulate the relevant microbiota-host interactions through targeted interventions. The current state-of-the-art suggests that the microbiota could offer relevant clinical applications for HIV and HPV diseases that might prove suitable to stratify the risk of HIV acquisition (reviewed in (176)), helping to the diagnosis of comorbidities (e.g., tuberculosis or anal dysplasia). This field might also advance the therapeutic options for HIV and HPV, including the development of new treatments or adjuvants through probiotics or postbiotics that could lead to more personalized medicine approaches, including targeting chronic inflammation (67), enhancing immune recovery (60), or facilitating HPV clearance (177). However, if we want to translate our current knowledge into clinical applications, we will have to overcome several methodological challenges, such as standardization of the methods to assess the species level and identify unknown microorganisms that represent today a significant fraction of the microbiota. Advancing culturomic approaches, microbiome-imaging techniques, multiomic integration, and validating the findings in hypothesis-driven experimental designs will also help the field to move forward. Finally, we will need to validate the conclusions from translational research in observational or interventional studies designed *ad hoc* to test previously generated hypotheses. While one decade of research has paved the road for investigating clinical applications of the microbiome in HIV and HPV infections, we face the challenge of learning how to harness the microbiome in medicine in the next years.

## Author contributions

EM: writing first draft. All authors: revision and writing of manuscript. All authors contributed to the article and approved the submitted version.
